# Global trends in bufalin application research for cancer from 2003 to 2022: A bibliometric and visualised analysis

**DOI:** 10.1016/j.heliyon.2024.e24395

**Published:** 2024-01-09

**Authors:** Donghao Tang, Yuejiao Feng, Jiahao Lu, Linlin Jia, Dongxiao Shen, Jing Shang, Teng Chen, Peihao Yin, Jinbao Chen, Jie Wang

**Affiliations:** a Shanghai Putuo Central School of Clinical Medicine, Anhui Medical University, Shanghai, 200062, China; b The Fifth Clinical Medical College, Anhui Medical University, Anhui, 230022, China; c Department of General Surgery, Putuo Hospital, Shanghai University of Traditional Chinese Medicine, Shanghai, 200062, China

**Keywords:** Bufalin, Cancer, Bibliometric approach

## Abstract

**Background:**

Bufalin, the main active ingredient of the traditional Chinese medicine huachansu, is used in the clinical treatment of colorectal cancer and has multiple effects, including the inhibition of migratory invasion, reversal of multi-drug resistance, induction of apoptosis and differentiation, and inhibition of angiogenesis.

**Methods:**

We collected relevant articles on bufalin from 2003 to 2022 using the Web Science platform, and analysed the information using VOSviewer, CiteSpace, and Microsoft Excel to categorise and summarise the publications over the past 20 years.

**Results:**

We collected 371 papers, with a steady increase in the number of articles published globally. China has the highest number of published articles, whereas Japan has the highest number of citations. Currently, there is considerable enthusiasm for investigating the anti-tumour mechanism of bufalin and optimising drug delivery systems for its administration.

**Conclusion:**

For the first time, we present a comprehensive overview of papers published worldwide on bufalin over the past two decades and the progress of its application in tumour therapy. We summarised the key authors, institutions, and countries that have contributed to the field and the potential of bufalin for the treatment of cancer. This will help other researchers obtain an overview of progress in the field, enhance collaboration and knowledge sharing, and promote future research on bufalin.

## 1 Introduction

Malignant tumours pose a significant threat to human health and safety. Emerging treatment methods and drug applications have improved the prognosis of tumour patients compared to the past [1]. Tumour recurrence and metastasis remain major challenges [2]. Currently, chemotherapy is one of the most important treatments available [3]. However, the efficacy of anti-tumour drugs is often influenced by their safety and resistance [4,5]. Many studies have demonstrated that the interaction between the tumour microenvironment (TME) and anti-tumour drugs is a critical factor in determining treatment outcomes [6]. Traditional Chinese medicine, which has a long history in China, plays an important role in suppressing tumour development [7]. In China, traditional Chinese medicine is commonly administered orally in the form of herbs, particles, or capsules, as well as through injections [8].

Numerous studies have been conducted on the Chinese medicinal monomers and their active components. One such monomer, bufalin, is found in the precious Chinese herbal medicine, ChanSu, and has a wide range of anti-tumour effects [9]. It is the main functional component of cinobufagin injections which has been used clinically in China [10]. The molecular formula of bufalin is C_24_H_34_O_4_, with a relative molecular mass of 386.56. Currently, physicians in China use cinobufagin, which contains bufalin, for the systemic treatment of mid-stage tumours [11]. It plays a crucial role in inhibiting cell migration and invasion, reversing multi-drug resistance, inducing apoptosis, and inhibiting blood vessel production [12]. Its mechanism involves multiple signal pathways, such as PI3K-AKT, HEDGENHOG, MAPK, JNK, WNT/Β-CATENIN, TGF-β/SMAD, integrated vegetarian signalling pathway, and NF-κB signalling pathway [13,14]. While there have been numerous reports on its anti-tumour mechanisms, not all of the mechanisms of action have been clearly and credibly proven. Bufalin has been shown to have inhibitory effects on various tumour cells, such as gastric, breast, colon, osteosarcoma, gallbladder, liver, lung, and pancreatic cancer [15]. Bibliometric analysis is a scientific and quantitative method used to assess published articles [16]. This paper outlines recent developments in this field. Our research focused on identifying the most influential publications, authors, journals, institutions, and countries. Additionally, we analysed the structure of cited references and journals. This section discusses the importance of understanding the theoretical basis of platform research, and how it can be explored using tools such as CiteSpace and VOSViewer [17].

**Fig. 1:**
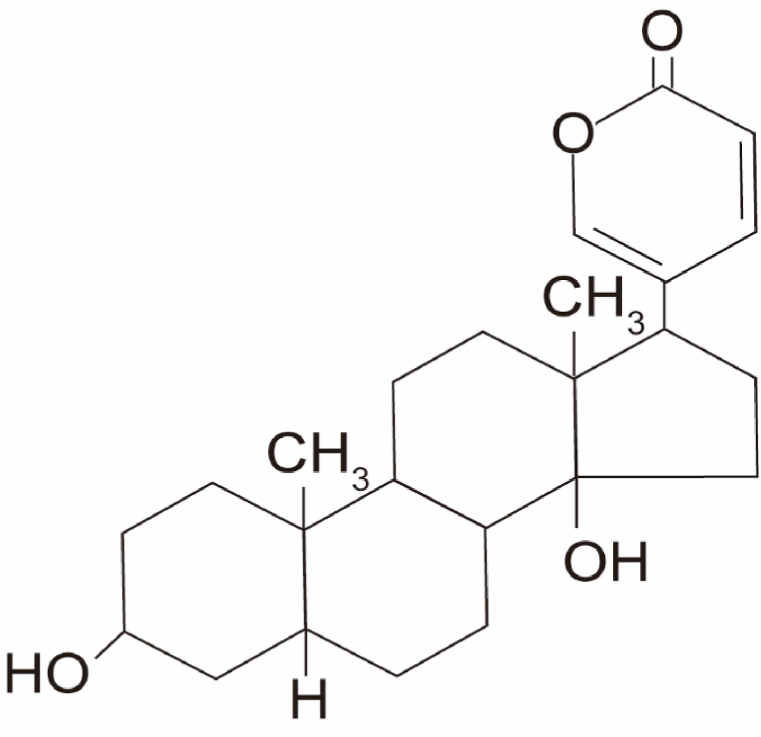
The chemical structure of bufalin.

Traditional Chinese medicine is a global medical resource. There have been many bibliometric studies of acupuncture [18], curcumin [19], artemisinin [20], and the Qingfei Paidu decoction in traditional Chinese medicine [21]. Despite the potential anti-tumour effects of bufalin, no bibliometric studies have been conducted on this topic. In this study, we utilised metrology to analyse and quantify the relevant articles published on bufalin over the past 20 years. We also employed a systematic review to thoroughly examine the relevant literature and the current research status of bufalin. Our findings provide valuable guidance for future scientific and clinical studies. Additionally, we have included a visual representation of the chemical structure of bufalin, as shown in Fig. 1.

## 2 Materials and methods

### 2.1 Date source and search strategy

**Fig. 2:**
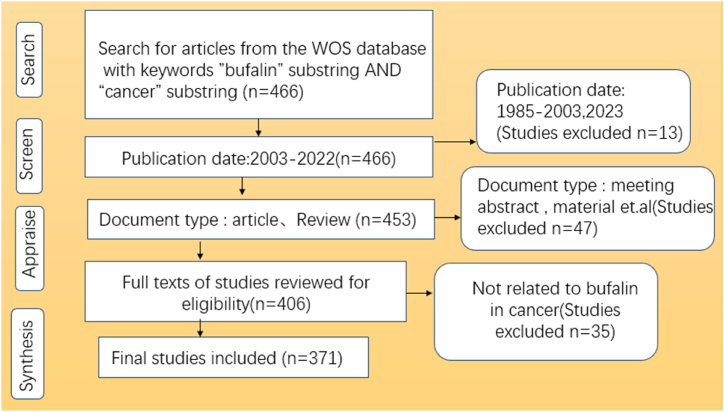
The inclusion criteria for paper selection.

This study extracted bufalin-related data and cancer-related data from the Web of Science Core Collection (WOSCC) database from 1 January 2003 to 31 December 2022. The retrieval method is illustrated in Fig. 2. We selected the following keywords: TS = (“Bufalin” OR ″ cinobufagin " OR “huanchansu”) AND TS = (“Tumour” or “Neoplasm” or “Tumours” or “Cancer “or “Cancers” or “Malignant Neoplasm” or “Malignancy or Malignancies” or “Malignant Neoplasms” or “Neoplasm, Malignant” “Benign Neoplasms” or “Benign Neoplasm” or “Neoplasms, Malignant” or “Neoplasms Benign” or “Neoplasm Benign” or “Neoplasia” or “Neoplasias”). This study utilised the WOSCC as the primary data source. To ensure the accuracy and integrity of the retrieved data, cinobufagin, which is mainly composed of bufalin, was added as a TS. The SCLEXPANDED and SSCI indices were selected for the search. The final search strategy retrieved 466 papers, which were then screened (Fig. 2). Finally, 371 valid studies were identified.

### 2.2 Analytical methods

Using CiteSpace 6.2.R2 and VOSviewer 1.6.16, we performed the data visualisation. CiteSpace, developed by Professor Chaomei Chen, is a software which can visualise networks among research hotspots and documents, as well as citation collaboration [22,23].

Two reviewers independently selected and extracted data from the most recent studies, including information on annual publications, countries, institutions, authors, keywords, journals, and citation frequency. After data collection, Microsoft Excel, CiteSpace, and VOSviewer were used as basic tools for visual analysis. Additionally, the online literature metrology analysis platform (http://bibliometric.com/) was used for further analysis.

## 3 Results

### 3.1 Number of global publications

**Fig. 3:**
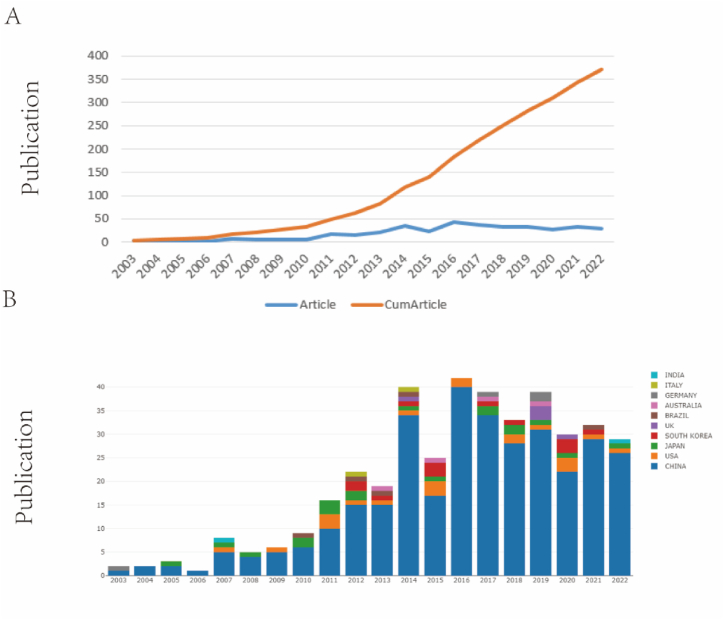
Annual trends in paper publication quantity.

We illustrate the timeline for the publications of bufalin in the tumour suppression research domain (Fig. 3A and B). There has been a steady increase in the number of articles on bufalin in oncology, with a significant increase between 2003 and 2014. The number of published papers remained stable in 2015, with 22 papers averaging more than 25. This indicates sustained interest among researchers studying bufalin in recent years.

### 3.2 Authors and co-cited authors analysis

**Fig. 4:**
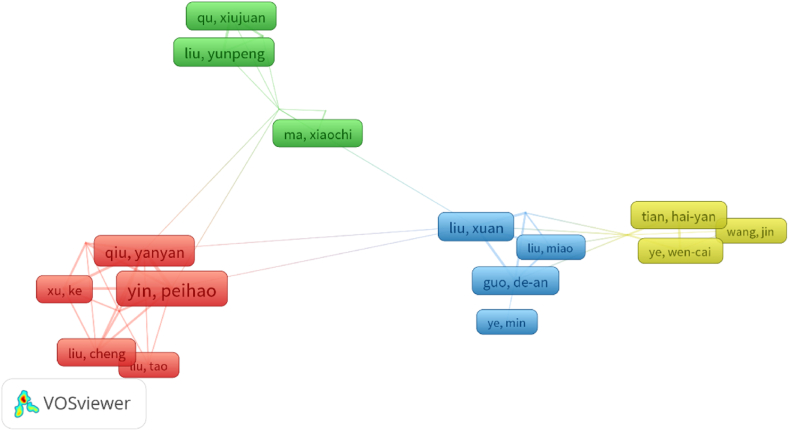
Bibliometric analysis of the author.

Studying the authors with the highest number of articles in a specific field has practical implications. First, it aids in identifying key leaders and experts in the field, the research findings of which may significantly influence development. Second, the output of these authors can serve as an important reference for other researchers and contribute to advancements in the field. Therefore, identifying the authors with the highest number of articles in a particular research area is relevant for assessing research dynamics and trends in the field. Through a literature analysis, notable scholars in the field of bufalin anti-tumour research were identified. Yin Peihao emerged as the most prolific author, publishing 16 papers between 2012 and December 2022 in Table 1. These papers have received 289 citations, with an average of approximately 18 citations per article. Liu Yunpeng had the highest number of citations per article, at approximately [48]. Fig. 4, which displays the author cluster analysis, reveals that Yin Peihao and Qiu Yanyan frequently collaborated on research projects. These scholars are affiliated with the Shanghai University of Traditional Chinese Medicine, and their research focuses on the impact of bufalin on reversing colorectal cancer drug resistance and inhibiting colorectal cancer metastasis.

**Table 1:** The top 10 Authors that contributed publications on bufalin.

Rank	Author	Documents	Citations	Average citations
1	Yin, Peihao	16	289	18
2	Chung,Jing-gung	15	411	27
3	Liu, Xuan	10	282	28
4	Meng, Zhiqiang	10	438	44
5	Qiu, Yanyan	10	191	19
6	Liu, Yunpeng	9	428	48
7	Zhang, Dong-mei	9	274	30
8	Guo, De-an	9	140	16
9	Qu, Xiujuan	8	314	39
10	Tian, Hai-yan	8	205	26
11	Wang,Yan	8	199	25
12	Ye,Wen-cai	8	205	26

### 3.3 Journals and co-cited journals analysis

**Fig. 5:**
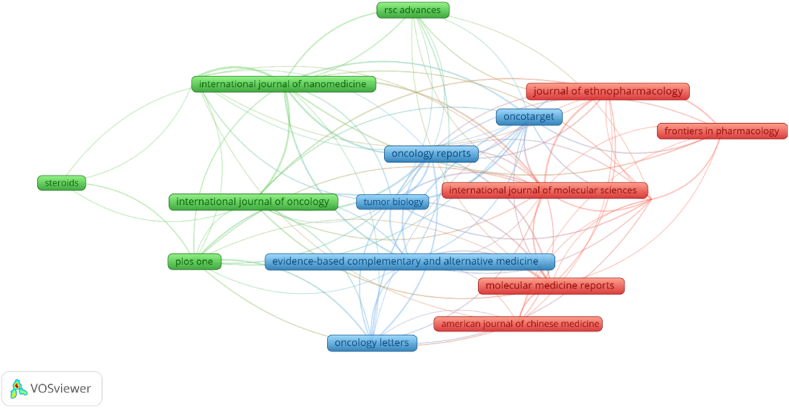
Journals of bufalin and tumor articles were published by vosviewer analysis.

**Fig. 6:**
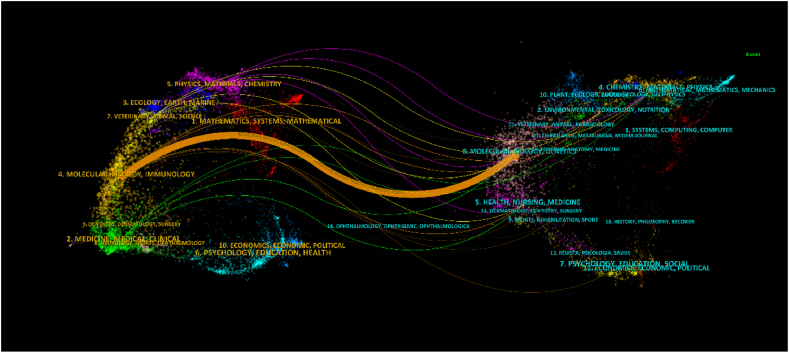
A double-plot overlay of articles citing bufalin and Tumor Research, with citing journals on the left, cited journals on the right, and straight paths representing citation relationships.

We analysed 371 articles from 175 journals. Fig. 5 illustrates the clustering of journals that published more than five articles on bufalin. Our analysis revealed that in the past two decades, comprehensive journals have published few articles on bufalin, with most of them being published in medical journals. Table 2 lists the top 10 journals ranked by publication volume, with *Oncology Reports* and *Oncology Letters* being the top publishers with over 10 articles on bufalin. According to the given information, 11 and 10 articles were published in these two journals, respectively. However, the average number of citations for the journal *Oncology Letters* was higher, suggesting that the quality of the literature published in this journal is superior and has garnered significant attention in the field of anti-tumour research on bufalin. This journal covers the entire field of medical oncology and published literature. Current literature highlights the potential of bufalin to reverse drug resistance in tumour cells and promote apoptosis. The molecular mechanisms underlying the inhibitory effects of bufalin on tumour growth have been the focus of most research, with many of the 10 ten journals in the field being open source. In recent years, open-source journals have played a significant role in promoting the use of bufalin in tumour research. However, there is still debate among academics regarding the best methods for accessing literature. However, the idea that scientists worldwide can access the latest research results for free is widely agreed upon. Fig. 6 presents a visual representation of the relationship between research topics in the fields of bufalin and oncology research. The dual map overlay shows the citing and cited journals, with the orange path indicating that studies published in *‘Molecular, Biology, Immunology'* journals are primarily cited in *‘Molecular, Biology, Genetics'*.

**Table 2:** The top 10 Sources that contributed publications on bufalin.

Rank	Source	Documents	Citations	Average citations
1	Oncology Reports	11	321	29
2	Oncology Letters	10	111	11
3	Oncotarget	9	264	29
4	Journal of Ethnopharmacology	9	236	26
5	International Journal of Oncology	9	210	23
6	Molecular Medicine Reports	9	108	12
7	Plos One	8	188	24
8	Evidence-based Complementary and Alternative medicine	8	177	22
9	International Journal of Pharmaceutics	7	198	28
10	International Journal of Nanomedicine	7	190	27

### 3.4 Institutions analysis

Table 3 displays the top 10 institutions based on the number of papers published, total cited frequency, and average cited frequency. All these institutions are located in China. Shanghai University of Traditional Chinese Medicine holds the first position on the list with 45 papers and 898 citations, followed by China Medical University with 35 papers, Shanghai Jiao Tong University with 21 papers, Fudan University with 19 papers, and Dalian Medical University with 17 publications.

**Table 3:** The Top 10 most Organizations in the field of Bufalin.

Rank	Organization	Documents	Citations	Average citations
1	Shanghai University of Traditional Chinese Medicine	45	898	20
2	China Medical University	35	909	26
3	Shanghai Jiao Tong University	21	391	19
4	Fudan University	19	680	36
5	Dalian Medical University	17	160	9
6	Chinese Academy of Sciences	16	202	13
7	Peking University	14	417	30
8	China Medical University Hospital	14	355	25
9	Asia University	13	379	29
10	Jinan University	13	273	21

**Fig. 7:**
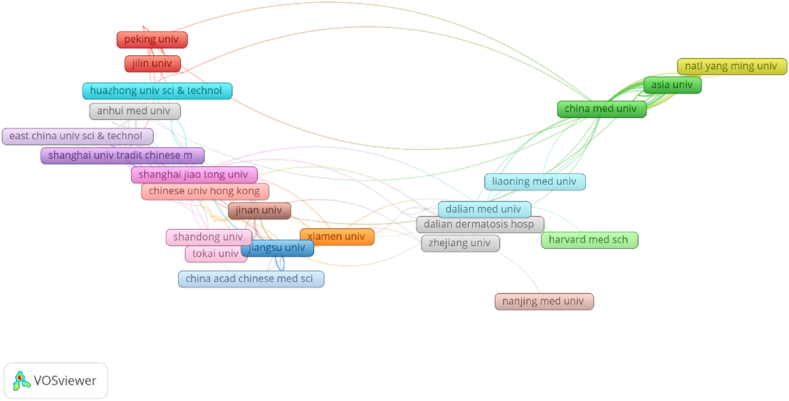
VOSviewer network visualizes the cooperation relationship diagram of different institutions.

Cooperation network analysis indicated that Shanghai University of Traditional Chinese Medicine has a significant impact on bufalin compared to other institutions. It was also observed that most of the institutions’ collaborations were limited to their respective countries, and international cooperation was minimal, as illustrated in Fig. 7. Additionally, network analysis revealed that China has been the primary contributor to research articles on bufalin in the field of anti-tumour medicine in recent years. Moreover, Fudan University has the highest average citation frequency, as depicted in Table 3.

China has made important advances in the field of bufalin anti-tumour. Below are some examples of specific research or advances in this area from some Chinese research institutions that have had a significant impact on the field: (1) Shanghai University of Traditional Chinese Medicine, whose research team has made some important advances in anti-tumour research on bufalin. The authors studied the anti-tumour effects of bufalin in animal models and explored its mechanism of action. These findings are expected to guide the clinical application of bufalin. (2) China Medical University: this research institute is a key institution in the research and development of bufalin. They conducted in-depth research on the molecular mechanisms of anti-tumour effects of bufalin. These discoveries provide a foundation for the study of bufalin and further promote research in related fields. (3) Shanghai Jiao Tong University: A research team from this university has made progress in developing a targeted drug delivery system for bufalin. They developed a nanoparticle-based drug delivery system for bufalin with improved drug delivery and targeting. This research aids in improving the effectiveness of bufalin and reducing its toxic side effects on healthy cells. The efforts and achievements of these research institutes have significantly impacted the research and application of bufalin in the field of anti-tumour therapy.

### 3.5 Countries analysis

**Fig. 8:**
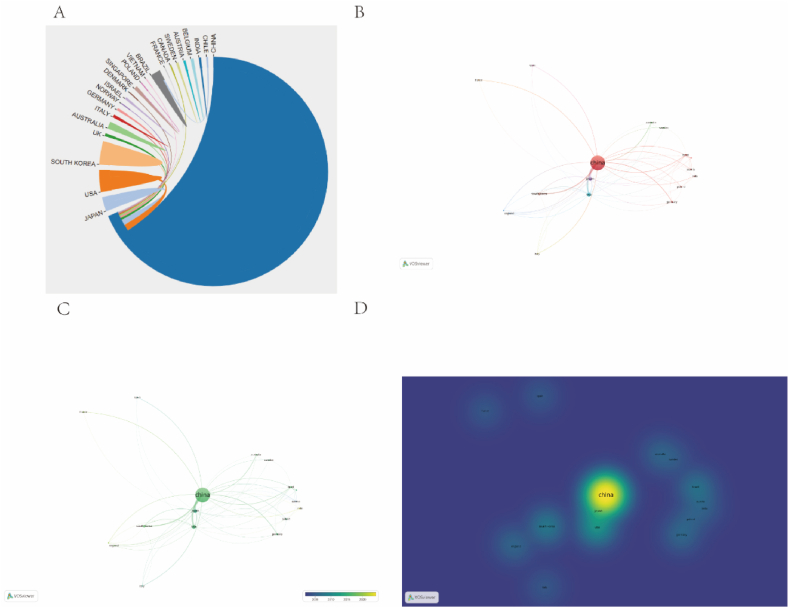
Overview of national publications. (A) Cooperation between countries. (B–D) National Cooperative Network.

A visual analysis of the number of publications in each country was conducted to analyse the distribution of research results in this field in different countries. The top five countries with the highest number of publications were counted using VOSviewer, and the results are presented in Table 4. To further analyse the countries with high productivity in this field, Table 4 lists the top five countries with the largest number of publications in this field. In this field, Chinese scholars contributed the most research papers with 327 publications, accounting for 88 % of the total number of papers published. However, the number of citations in the literature was relatively low. The United States has published 24 papers with 913 received citations. Japan was the first country to publish articles on the effects of bufalin on tumour [24,25]. Japanese articles on bufalin anti-tumour research are often of high scientific quality and reliability and include in-depth studies on the molecular mechanisms, anti-tumour effects, and clinical applications of bufalin. These results are often recognised and widely cited in peer-reviewed journals. Japan has the highest number of citations per article, with 20 papers receiving 891 citations. The average number of citations per article is approximately 45. In our study, we utilised an online bibliometric platform to analyse the collaborative network visualisation countries (Fig. 8A). Our findings indicate that China has the highest positive influence among all countries, followed by the United States, South Korea, and Japan. We also observed that China and Japan have the most cooperative relations, whereas the United States has established cooperative relations with most countries worldwide. To conduct the visual analysis, we employed the VOSViewer software, as depicted in Fig. 8B. Historically, China, the United States, and Japan have been the dominant countries publishing research in this field. However, in recent years, there has been a noticeable increase in research in India and France, suggesting that these countries have significant potential in this area (Fig. 8C). The density map (Fig. 8D) provides a clear visual representation of the number of publications per country.

**Table 4:** The Top 10 most Countrys in the field of Bufalin.

Rank	Country	Documents		Citations	Average citations
1	China	327		7594	23
2	USA	24		913	38
3	Japan	20		891	45
4	South Korea	11		207	19
5	Brazil	6		171	29

### 3.6 Keywords analysis

**Fig. 9:**
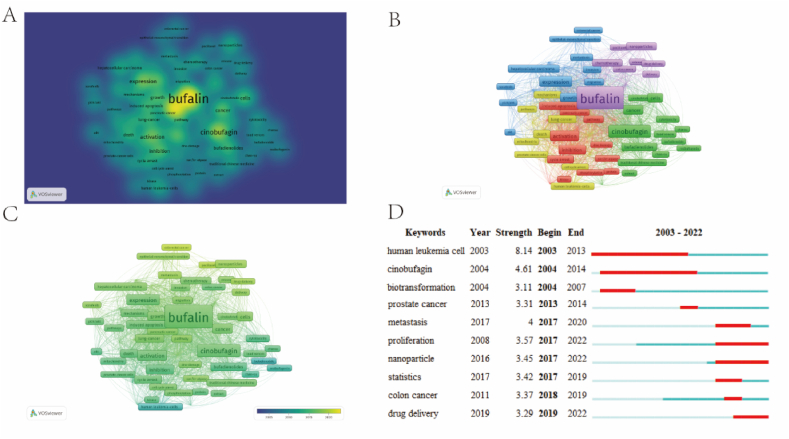
Keyword analysis. (A–C) Draw keyword co-occurrence clustering diagram (D) Visual map of the top10 keywords with the strongest citation bursts in the bufalin in cancer field.

Our analysis revealed that the keywords ‘bufalin' and 'apoptosis' were the most frequently used keywords in the literature, indicating that the induction of apoptosis in tumour cells by bufalin has garnered significant attention from researchers (Fig. 9A–C). Apoptosis generally occurs through three pathways: the mitochondrial, endoplasmic reticulum, and death receptor pathway [26]. The apoptosis-inducing effect of bufalin is a hot topic in current research, and the mechanism by which bufalin induces apoptosis in tumour cells often involves multiple pathways. Studies have shown that bufalin can induce oxidative stress in glioma cells, alter the homeostasis of intracellular ions, disrupt the permeability of mitochondria, increase the expression of caspase-3, and cause apoptosis in tumour cells [27]. In addition, bufalin acts on human tongue cancer cells; the protein levels of caspase-3 increase after 2 days, and caspase-9 is expressed after 3 days [28].

This study aimed to identify research hotspots, trends, and frontier dynamics within a certain period by analysing the most frequently detected keywords. Ten keywords were identified, all of which had a bursting strength greater than 3, as shown in Fig. 9D. The analysis revealed that Bufalin has been extensively studied in the context of haematological tumour cells, particularly in the early stages. Overall, haematological tumour cells are a popular area of study. Drug delivery systems for bufalin have also been a focus of research. Through the development of drug delivery systems such as nanoparticles and carriers, the delivery effect and targeting of bufalin can be improved, and the toxic side effects on normal cells can be reduced. There has been an increase in research into the combination of bufalin and new nanomaterials. This combination has the potential to reduce the side effects of bufalin and can be used as a drug delivery system that specifically targets tumours [29,30]. Additionally, this combination may enhance the induction of apoptosis in tumours [31].

### 3.7 Cited articles analysis

Table 5 presents the 10 most-cited articles, with *Cancer Science* having the highest number of published studies. One of them was the study conducted by Meng et al., in 2009, titled ‘Pilot study of huachansu in patients with hepatocellular carcinoma, non-small Cell Lung Cancer, or Pancreatic Cancer' (n = 90). The study was a collaboration between Fudan University Cancer Hospital and the University of Texas M. D. Anderson Cancer Centre and concluded that huancansu was well tolerated. A clinical trial conducted in China confirmed the safety and tolerability of a drug containing bufalin as the main ingredient, which provided stable disease in subgroups of patients when used alone [32]. Subsequently, there has been a significant increase in the literature on bufalin, with nine additional articles published between 2003 and 2011, citing previous studies. Among these ten articles, seven explored the effect of bufalin on the induction of apoptosis in tumour cells. Han et al. conducted an animal experiment and found that bufalin up-regulates the expression of the apoptosis-regulating gene bax, resulting in resistance to orthotopic transplantation of human hepatocellular carcinoma in nude mice [33]. Qi et al., through an in vitro study, confirmed that bufalin induces apoptosis in human hepatocellular carcinoma cells through mitochondria-mediated pathways [34]. Furthermore, other studies have showed that bufalin induces apoptosis in ovarian [35], gastric [36], and prostate cancer cells [37]. In summary, these studies suggest that bufalin has significant potential as it has shown inhibitory effects on various tumour cells. Further research is required to translate these findings into clinical practice.

**Table 5:** The top 10 most co-cited references related to Bufalin.

Author	Citations	Title	Journal	Year
MengZq	90	Pilot Study of Huachansu in Patients With Hepatocellular Carcinoma, Nonsmall-Cell Lung Cancer, or Pancreatic Cancer	Cancer-am Cancer Soc	2009
Yu Ch	84	Apoptotic signaling in bufalin- and cinobufagin-treated androgen-dependent and -independent human prostate cancer cells	Cancer Sci	2008
Yeh Jy	78	Effects of bufalin and cinobufagin on the proliferation of androgen dependent and independent prostate cancer cells	Prostate	2003
Li D	74	PI3K/Akt is involved in bufalin-induced apoptosis in gastric cancer cells	Anti-cancer Drug	2009
Qi Fh	74	Bufalin and cinobufagin induce apoptosis of human hepatocellular carcinoma cells via Fas- and mitochondria-mediated pathways	Cancer Sci	2011
Qi Fh	74	Antitumor activity of extracts and compounds from the skin of the toad Bufo bufo gargarizans Cantor	Int Immunopharmacol	2011
TakaiN	55	Bufalin induces growth inhibition, cell cycle arrest and apoptosis in human endometrial and ovarian cancer cells	Int J Mol Med	2008
Xie *Cm*	55	Bufalin induces autophagy-mediated cell death in human colon cancer cells through reactive oxygen species generation and JNK activation	Free Radical Bio Med	2011
Han Kq	52	Anti-tumor activities and apoptosis-regulated mechanisms of bufalin on the orthotopic transplantation tumor model of human hepatocellular carcinoma in nude mice	World J Gastroentero	2007
Jiang Yt	51	Effects of bufalin on the proliferation of human lung cancer cells and its molecular mechanisms of action	Cytotechnology	2010

## 4 Discussion

Malignant tumours pose a serious threat to human health, with an increasing global incidence in recent years. It is now the second leading cause of death after heart disease [38]. Consequently, the world is increasingly focusing on the potential of natural medicines in combating cancer. Many of these medicines have been successfully used in cancer treatment [39]. The combination of traditional Chinese and Western medicines shows great promise as a treatment method [40]. Although the inhibitory effect of bufalin on tumours was not reported until 1999 [41], the number of publications on this topic continued to increase until 2014. Since then, the annual publication volume has gradually stabilised, and more than half of the relevant research publications were published after 2014, indicating rising research enthusiasm and trends. Bufalin has many effects on tumour growth. Bufalin is thought to have anti-tumour activity and may inhibit the proliferation of tumour cells. This effect may be achieved by interfering with DNA synthesis or disrupting the chromosome structure of cancer cells [42]. Therefore, Bufalin can induce apoptosis, which is a programmed cell death process, in tumour cells. Apoptosis is a part of the body's regulatory mechanism that can help remove abnormal or damaged cells [13]. It is important to note that the exact mechanism of action of bufalin may vary among different types of tumours and cell lines. In addition, the use and efficacy of bufalin are still being researched, and further clinical studies and experimental validation of its exact effects and effectiveness are required. In this information-rich era, bibliometric analyses can help researchers to quickly understand the background, current status, hotspots, and research directions in a given field. However, to date, no study has provided a bibliometric overview of bufalin in the field of anti-tumour. This article visually describes the development process, research hotspots, and evolutionary trends of bufalin and provides a comprehensive analysis using knowledge maps to help researchers build collaborative relationships and work together to conduct more research and advance the discipline.

Based on a literature analysis spanning 20 years, it was found that the majority of research on bufalin and its relation to tumours originated in China, followed by the United States, Japan, and South Korea. The 10 ten institutions with the most published papers were all in China, which can be attributed to the country's rich history and culture surrounding traditional Chinese medicine, as well as its extensive research on natural medicines. In addition, Traditional Chinese medicine has a history of thousands of years in China. Notably, the discovery of artemisinin was acknowledged with a Nobel Prize. The Chinese government places a significant emphasis on the advancement of traditional Chinese medicine and provides funding and technical support for research in this field. In terms of molecular mechanism research, Chinese scientists have conducted in-depth studies on bufalin, revealing the mechanism of its effects on tumour cell growth, apoptosis and metastasis. These studies provide an important theoretical basis for the further development of bufalin as an anti-tumour drug. In addition, Chinese researchers have actively explored the combined application of bufalin and other Chinese medicinal compounds to find ways to improve anti-tumour effects and reduce toxic side effects. This comprehensive approach to the use of traditional Chinese medicine has made a unique contribution to anti-tumour studies of bufalin, providing important scientific support for its development and application as an anti-tumour drug. The Shanghai University of Traditional Chinese Medicine is a leading institution in this area and has published a substantial number of articles. Yin Peihao had the highest number of publications. A significant amount of the theoretical groundwork for the clinical use of traditional Chinese medicine, specifically the active monomer bufalin, was provided by Yin Peihao. Among the journals that have published the most articles on this subject, *Oncology Reports* stands out because of its comprehensive coverage of basic tumour research with a focus on tumorigenesis and metastasis. Additionally, other journals such as *Oncology Letters*, *Oncotarget*, *Journal of Ethnopharmacology*, and *International Journal of Oncology* have published several relevant papers. Conversely, the impact factors of published journals on traditional Chinese medicine, including bufalin, are generally low and mostly published in China. Improving the influence of Chinese journals can aid the dissemination of traditional Chinese medicine. Cluster and word burst analyses were conducted on the literature and keywords related to bufalin, revealing that it exerts anti-tumour effects through a complex molecular signalling network. In recent years, there has been growing interest in studying the TME and nanomaterials [7]. Several studies have highlighted the significance of the TME in tumour development [43]. Currently, a hot topic of interest is investigating the correlation and interplay between traditional Chinese medicine and anti-tumour therapy in the TME [8]. Chinese medicine has been used to treat many-cancer patients in China [39]. Several studies have demonstrated that the antitumor mechanism of bufalin is associated with the TME, including macrophages [44], neutrophils [45], fibroblasts [46], and vascular endothelial cells [47]. Our previous studies also revealed that bufalin can regulate the polarisation of macrophages by targeting the SRC-3/MIF pathway, leading to anti-tumour effects [48]. However, treating patients with advanced tumours is complex, and a single treatment may not produce a sufficient curative effect. Bufalin has been shown to increase the efficacy of chemotherapeutic drugs in several studies. In a study conducted by Ying et al., bufalin was combined with gemcitabine and applied to three pancreatic cancer cell lines (Bxpc-3, Mia PaCa-2, and Panc-1) in vitro. A previous study found that this combination promoted the apoptosis of tumour cells [49]. Resistance to chemotherapy and its associated side effects have become the primary barriers to improving the efficacy of cancer treatment. Recently, the use of natural compounds in combination with standard chemotherapeutic agents has proven to be an effective therapeutic approach. Bufalin has been shown to reduce the required dose of chemotherapeutic agents and decrease their toxicity [50].

Furthermore, the focus of bufalin research has shifted from its traditional use as a combined chemotherapeutic drug to its potential use as a combined targeted drug. A study conducted by Jung et al. demonstrated that a combination of Sorafenib and Bufalin acutes caspase signalling pathways, reactive oxygen species, and mitochondria in vitro, which enhances apoptotic cell death in NCI–H292 lung cancer cells [51]. However, their high toxicity and short half-lives limit their potential as effective anticancer drug [52]. Additionally, most studies have been conducted in vitro, and, there have been only few in vivo evaluations of its therapeutic effects, hindering its development. To address this issue, researchers are focusing on reducing the side effects of bufalin to further develop it as a potential anticancer drug [53]. Recent studies have shown that nano-preparations of bufalin, such as nanoparticles, liposomes, micelles, polymers, and nanogels, can overcome biological defects, improve its stability in vitro and in vivo, and increase its cellular bioavailability [54]. As a result, the use of bufalin-combined nanomaterials has gained attention in recent years, highlighting bufalin as a research hotspot and frontier in the field of tumour therapy. Despite these limitations, the development and application of bufalin nanomaterials for tumour treatment show promising potential. The current trend in tumour treatment involves a diverse range of approaches, with drug combination therapy being a promising area for future research. In summary, bufalin, along with traditional Chinese medicines that contain bufalin as their main active ingredient, has exhibited significant potential as an anti-tumour therapy.

Despite the progress made in research on bufalin in the field of oncology, there are still many challenges and unresolved issues. For example, the exact mechanism and target of action of bufalin are not yet fully understood. The sample size of clinical studies is relatively small, and larger studies are needed to verify its efficacy and safety. Therefore, the current status of bufalin research requires continuous in-depth exploration and further studies. (1) Conducting preclinical studies and animal experiments: Before conducting clinical trials, additional preclinical studies and animal experiments should be conducted to assess the safety and efficacy of bufalin. These include studies on drug metabolism, pharmacokinetics, and toxicology. (2) Exploring diversified applications of bufalin: In addition to the treatment of tumours, the application of bufalin in other fields should also be explored. For example, their potential roles in immunotherapy, aging, and neurodegenerative diseases have been investigated. (3) Strengthening collaboration and knowledge sharing: In Bufalin's research, strengthening collaboration and knowledge sharing can accelerate research progress. Researchers can promote interdisciplinary collaboration and sharing of data and research results to promote future research on the bufalin spirit.

## 5 Conclusion

This bibliometric study analysed institutions, countries, authors, journals, hotspots, and future developments of bufalin in the field of cancer research over the past 20 years. We anticipate an increase in research on bufalin in the field of tumours in the future.

## 6 Limitations

First, the data were extracted solely from WoSCC, and articles might be overlooked in other sources such as PubMed and Scopus. Therefore, the results of the present study may have been affected. Second, we analysed complete annual data; the time-frame of the included publications was limited to 2003 and 2022, and articles published in 2023 were excluded. Therefore, information regarding bufalin in 2023 may have been missed. Finally, the literature we retrieved was limited to English, and some articles in other languages may have been omitted.

## Funding

This work was supported by the 10.13039/501100001809National Nature Science Foundation of China (81973625), China; the foundation of the 10.13039/501100012166National Key Research and Development Program of China (2019YFC1316000), China;the foundation of the Shanghai Key Medical Specialty Construction Project (ZK2019B18), China; the foundation of the Clinical Specialized Disease Construction Project of Shanghai Putuo District Municipal Health Commission (2019tszb01), China; and the foundation of the 10.13039/501100002947Anhui Medical University Postgraduate Scientific Research and Practice Innovation Project (YJS20230098), China.

## Data availability statement

Data sharing does not apply to this paper; the data currently used in this study is from Web of Science, so access to the data requires permission through Web of Science.

## CRediT authorship contribution statement

**Donghao Tang:** Data curation, Conceptualization. **Yuejiao Feng:** Data curation. **Jiahao Lu:** Software, Resources. **Linlin Jia:** Investigation. **Dongxiao Shen:** Visualization. **Jing Shang:** Formal analysis. **Teng Chen:** Supervision. **Peihao Yin:** Supervision. **Jinbao Chen:** Writing – review & editing, Writing – original draft, Funding acquisition. **Jie Wang:** Writing – review & editing, Writing – original draft, Validation, Funding acquisition.

## Declaration of competing interest

The authors declare that they have no known competing financial interests or personal relationships that could have appeared to influence the work reported in this paper.
